# Examining Chinese Users’ Feedback Comments on HIV Self-testing Kits From e-Commerce Platforms: Thematic and Content Analysis

**DOI:** 10.2196/38398

**Published:** 2022-11-30

**Authors:** Miao Liu, Yi Zhu, Haiqing Gao, Jialing Li

**Affiliations:** 1 School of Journalism and Communication Beijing Normal University Beijing China

**Keywords:** HIV self-testing, Chinese, feedback comments, e-commerce platforms

## Abstract

**Background:**

HIV self-testing is preferred by many Chinese people for its convenience and confidentiality. However, most studies on HIV self-testing (HIVST) uptake in China overfocused on men who have sex with men and overrelied on obtrusive methods such as surveys and interviews to collect data.

**Objective:**

We aimed to explore Chinese HIVST kit users’ authentic experience via their feedback comments posted on e-commerce platforms using an unobtrusive approach.

**Methods:**

In total, 21,018 feedback comments about buying and using HIVST kits posted on Chinese e-commerce platforms (Tmall and Pinduoduo) were collected. An inductive thematic analysis based on a random sample of 367 comments yielded several thematic features. These thematic features were developed into coding categories for a quantitative content analysis of another random sample of 1857 comments.

**Results:**

Four themes were identified in the first study, including the expression of positive and negative emotions after and before getting the test, respectively, calling for living a clean and moral life in the future, comments on the sellers and HIVST kits, and the reasons for buying HIVST kits. The results from the second study suggested that there were significant associations between different platforms and several thematic features. Nearly 50% of the comments were related to the product itself and the disclosures of HIV-negative test results. More than 25% of the comments showed users’ feelings of gratefulness after receiving negative test results such as “thank heavens for sparing my life.”

**Conclusions:**

The results suggested that Chinese users relied on HIVST kits to reduce and prevent HIV infection, while they also considered HIV infection a punishment related to moral violation such as being sexually promiscuous. The traditional Chinese health belief that health is influenced by one’s morality still persists among some Chinese users. Many users also lacked appropriate knowledge about HIV transmission and self-testing kits.

## Introduction

### Background

In recent years, there has been an upsurge of HIV infection rates in China, particularly among young people [1]. Early detection of HIV is of vital importance to AIDS intervention and treatment. Among different HIV testing options, HIV self-testing (HIVST), also known as home testing, is more preferred by young people for its convenience, confidentiality, control over disclosing the status to others [2], and social acceptability [3]. HIVST is a process whereby individuals collect their specimens such as oral fluid and blood to perform and interpret HIV rapid diagnostic tests themselves in private [4,5]. This procedure was recommended by the World Health Organization as an option to expand HIV testing coverage [6]. A recent study has shown good consistency between HIVST kit results and HIV antibody detection results [7]. There is a potential of using HIVST to reach the population at high risk such as sexually active young adults who preferred HIVST kits to traditional clinical tests for the accessibility and speed of results of HIVST kits [8].

Many pilot studies showed that HIVST received high acceptability for populations at risk of HIV infection in multiple countries such as Kenya [9], Canada [10], and the United States [11] because it is easy to use and could address stigma-related barriers to testing [9,12,13]. However, HIVST may result in risks in some resource-limited settings and bring about concerns with regard to policies, ethics, privacy issues, users’ safety, and public health issues such as the lack of linkage to care or failure to detect accurate HIV infection [4-6]. Although HIVST has its drawbacks, it expands HIV testing to a wider population, provides preliminary HIV knowledge [6], and contributes to informed sexual decision-making [4,12].

To lower the increasing HIV infection rate in China, the State Council of China issued Thirteenth Five-Year Plan (2017-22) for HIV and AIDS prevention and control in which the government encourages the expansion of HIVST by exploring [14] the strategies to promote HIVST through selling self-testing kits in pharmacies and sellers on the web. The Chinese Center for Disease Control and Prevention and Chinese STD and AIDS Association have also been promoting HIVST by providing HIVST-related information through different channels [15]. In 2019, the Chinese Center for Disease Control and Prevention published the “Manual of AIDS Self-testing Guidance” [16] to provide at-risk populations with informational support.

There are robust HIVST studies examining high-risk populations such as men who have sex with men (MSM) in China [7,17-19]. Han et al [17] found that self-testing among MSM was positively related to being married, having ≥6 male anal sex partners in the past 3 months, and having HIV tested within 12 months. Another study found that older age and marriage could be barriers for HIVST uptake, and higher educational levels and more HIV and AIDS knowledge are positive predictors of HIVST uptake among MSM [7]. In addition, administering HIVST among MSM was also preferred by students and those who have a junior college degree or higher and had anal intercourse with men in the past 6 months [20].

### Objectives

Overall, current HIVST studies in China have been overemphasizing MSM while ignoring a more general population who use HIVST. However, the largest group that uses HIVST kits in China might not be MSM. A recent study examined the profiles of those who purchased HIVST kits on the web and found ≥57% of the web-based buyers were heterosexual [21]. In addition, it is found that web-based buyers of HIVST kits tended to have higher education and upper-middle income and to be unmarried young men [21]. It should be noted that HIVST studies in China have overwhelmingly adopted obtrusive methods such as survey [7,17-19,21] and interview [12,22] in which those who took HIVST were approached by researchers or they were aware of their status as study participants. Because of HIV stigma in Chinese society [23], participants who completed the survey or got interviewed may not disclose truthful information about their self-testing experiences. In addition, due to the limitations of survey and interview methods, those studies discussed earlier have seldomly examined emotional responses and other sociopsychological factors for those who took HIVST. Taken together, we aimed to adopt an unobtrusive approach [24] to explore HIVST kit users’ authentic experiences of administering self-testing based on the web-based comments posted under HIVST kits sold on e-commerce platforms. This approach of examining user-generated health-related information is referred to as infodemiology, which helps to understand user knowledge, attitudes, behaviors, and information consumption [25-27]. Infodemiological approaches examined health communication and information distributed on the internet such as characteristics of health websites or consumers’ health informatics [25,28]. Infodemiological approaches have been used widely in health areas [29] to inform public health and policy and obtain user-generated opinions. Users’ feedback comments on health-related devices were used as textual data in infodemiological studies on Amazon customer reviews of direct-to-consumer hearing devices [28] and penile clamps [30]. However, there are few infodemiological studies on feedback comments for using HIVST kits in China. It is important to explore how user-generated comments on Chinese e-commerce websites reflect HIVST users’ emotional reactions, the reasons for buying HIVST kits, and concerns with their health status. More importantly, those comments are generated by a more general population in China instead of only MSM. As a result, this infodemiological study expanded on the current HIVST studies in China and provides a broader picture of Chinese HIVST kit users by examining their authentic user experience based on their feedback comments.

## Methods

### Overview

This research included 2 studies examining HIVST kit feedback comments on e-commerce platforms. The first study was an inductive thematic analysis to identify patterns from qualitative data to generate unanticipated insights [31]. Owing to the limited number of studies on the user experiences of HIVST kits, there is little evidence that can be used to derive specific research questions, so inductive thematic analysis was adopted to provide a preliminary picture of HIVST kit users in China. Themes identified from the comments on HIVST kits in the first study were discussed and then served as the coding categories for the second study, a quantitative content analysis. Quantitative content analysis is frequently used to analyze media messages to explore the patterns in message contents [32]. In the second study, we aimed to explore more specific questions raised from the first study based on a larger sample of comments to understand HIVST kit users’ profiles and their responses after purchasing HIVST kits.

### Data Selection and Collection

Feedback comments of HIVST kits from Pinduoduo and Tmall were selected for analyzing the HIVST users’ experiences. Tmall and Pinduoduo were chosen because both platforms have a huge number of users in China, but they have a different target market. Tmall is an e-commerce platform for top-quality branded products that guarantees all goods on the platform are authentic [33] and had 500 million users by January 2021 [34]. Pinduoduo is a rapidly growing e-commerce platform and had 867 million active buyers in 2021 [35]. Most Pinduoduo users are price-sensitive customers who live in lower-tier cities in China [35].

Data were collected in early December 2021. We searched the keyword “hiv test” in the platforms and identified the top 10–selling HIVST kits at Tmall mobile app and top 15–selling HIVST kits on the Pinduoduo mobile app based on their sales volume within the past month of data collection. The rationale to choose the top-selling products is that, usually, best-selling products generate most feedback comments. There were relatively fewer comments for each HIVST kit posted on Pinduoduo than Tmall, so more HIVST kits sold on Pinduoduo were selected to obtain enough comments. The HIVST kits sold on Tmall or Pinduoduo are either blood based or saliva based. All the HIVST kits discussed in this paper have passed China’s National Medical Device Registration Approval.

Feedback comments are defined as information including texts, signs, and emoticons posted publicly on Tmall and Pinduoduo’s comment section by HIVST kit users in this study. There were several comments such as “This user thinks this product is really good and gives a five-star rating” or “This user does not leave any comment,” which were automatically generated by the platforms while data were retrieved. These comments were excluded from the data corpus because they yielded little implication for our study and were not made by real commenters. Duplicated comments for a particular kit from the same commenter posted during the same period were also removed from this analysis because they might be fake reviews. The final data corpus pool included 21,018 comments (Pinduoduo: n=4989; Tmall: n=16,029).

### Ethical Considerations

All the comments retrieved from Pinduoduo and Tmall were posted publicly on the comment sections and were accessible to everyone visiting these 2 platforms. All personally identifiable information (such as commenters’ usernames in these platforms and names of locations mentioned in comments) included in the comments was removed from the analysis to protect commenters’ anonymity and privacy.

## Results

### Study 1: Thematic Analysis

#### Overview

A sample of 192 comments were randomly selected from the overall data corpus for thematic analysis. We followed the procedure of thematic analysis as suggested by Braun and Clarke [31] to generate initial codes, sort those codes into themes, review themes, and define themes. We then read another sample of 175 comments from the overall data to ensure no new themes appear [36].

Four major themes were identified from 367 comments posted on Pinduoduo (n=81) and Tmall (n=286), including the expression of positive and negative emotions, calling for living a clean and moral life, comments on the sellers and HIVST kits, and the reasons for buying HIVST kits. These themes are not mutually exclusive, so a single comment may include all 4 themes.

#### Expression of Positive and Negative Emotions

Under this theme, HIVST kit users expressed their positive emotions specifically focusing on gratefulness and relief after getting their negative test results, their negative emotions before getting the test, and their hope for others to get negative test results and stay healthy. In most cases, commenters tended to express their gratefulness to the heavens such as “Thank heavens and I will be definitely more cautious about my sexual activities,” while several commenters express their gratefulness to many targets. For example, 1 user commented as follows:

At last, I will thank that scumbag guy for not killing me. Thank heavens for sparing my life. Thank heavens for not killing me. Thank [the brand of HIVST kits].

In addition, comments expressing positive emotions such as relief were observed frequently:

Thank heavens for not killing me. Now I finally feel relief.

Some commenters expressed negative emotions before the test, such as fear and guilt, and positive emotions (gratefulness and relief) after the test, in a single comment, for example:

I will never forget the feeling at the day while waiting for shipping [of the HIVST kits] that I would rather be dead. Once recalling this, I was trembling all the time. Luckily, the result turned to be good. Take 20 minutes [to conduct HIVST] to get peace for the eternal life. Thank heavens. If you are afraid of [getting HIV]. Don’t panic. Don’s trust information on the Internet which is exaggerated. Please get [HIV] test.

I am looking at the reagent paper and thinking about my life for a while. The result is negative and I feel I get a rebirth inside. Recalling that experience in a few months ago, it might be the most regretful thing I did in my whole life. I would never think that I was associated with “AIDS” a few months ago. AIDS had deeply influenced my life in the past few months, I was bothered by different kinds of fear and worries inside. [AIDS] just surrounded me entirely and almost suffocated me.

I have been scared for a few months. It’s like I had been living in a hell in the past few months. After the test today, I finally feel relief. I will live a clean and moral life in the future and not feel worried any longer.

In front of [sexual] temptation, you just could not make any reasonable decision. Previously I committed such a sin and I was so frightened. I am too young and have no children. If I really got shot [to get AIDS], I deserved it. Luckily, thanks for heaven’s mercy to spare my life. Since this event, I feel so grateful and I will be a good person.

In addition to gratefulness and relief, HIVST kit users also expressed their hope for both themselves and other HIVST users to receive negative results. Two users commented as follows:

Thank heavens for not killing me. I hope the test result is accurate. I hope everyone who purchases this [HIVST kits] gets a negative test result!!!

If you found yourself getting infected earlier, you can get yourself treated earlier. I hope all of you just have a false alarm! Hope all of you [have] negative [test results]!

#### Calling for Living a Clean and Moral Life in the Future

This theme features calling for living a clean and moral life (*Jieshenzihao* in original Chinese comments) in the future with greater self-control for users themselves and other HIVST kit users. Commenters made promises to resist promiscuous lifestyles and made a determination to live a self-disciplined life. The following are a few examples:

I got a box of oral fluid test kits and a box of blood test kits...both test results are negative. I hope the test results are accurate. I will live a clean and moral life so diseases [HIV] will not attack me. I posted this comment here as a reminder to myself! And I shared this (comment) with everyone!

AIDS-phobia is so scary. I was so scared while waiting for the test result. I was too young to know how harmful it is when I had these risky acts. I could not fall into sleep these days [because I was] imagining how I should deal with my family members if I really got AIDS. Thank heavens for not killing me. If I was going out for some promiscuous acts in the future, may thunders strike me down.

Other HIVST kit users shared their experiences as a warning to raise HIV awareness and to call for others to resist promiscuous lifestyles:

I am warning everyone here. Never live promiscuously. You must live a clean and moral life and be careful about your health. I hope everyone gets a bright future, and I will start to live my new life.

After this experience, I have learned the importance of living a clean and moral life and will no longer fool around my own life...I hope everyone can live a clean and moral life and don’t fool around with your own life so easily.

#### Comments on the Sellers and HIVST Kits

Under this theme, HIVST kit users provided either positive or negative evaluations for web-based sellers and HIVST kits. As for their web-based shopping experiences with sellers on these e-commerce platforms, most comments were concerned with privacy and convenience about purchasing HIVST kits on the web, which is consistent with the previous findings on the advantages of using HIVST for the sake of privacy and convenience [18,22,37]. For example, some commenters made a comparison between using HIVST kits and visiting the clinic for a test and showed their preference for the former:

I feel that a heavy stone was finally removed from my heart. This reagent paper was amazing to protect me from awkwardness for visiting a clinic.

The price is really reasonable and much cheaper than visiting a clinic. The [shipping] speed is really fast. I bought it today and it will arrive tomorrow. Shipping service is really good.

Cheap and useful. Accuracy is as high as testing at a hospital.

An interesting observation from these comments is that few HIVST kit users complained about the cost of buying kits. Unlike previous findings based on MSM samples in which high cost is one of the top barriers for using HIVST [18,22], some users in this study even considered HIVST kits sold on e-commerce platforms to be very cheap. One explanation is that web-based buyers of HIVST kits tended to have upper-middle income. This may result from the demographic differences between the general population examined in this study and MSM samples in previous studies.

There are comments addressing how patient the customer service representatives were. Some buyers posted positive comments about sellers for fast delivery and discreet shipping to protect their privacy. Because of HIV stigma in China [38], those who purchased HIVST kits on the web were worried about their privacy, so discreet shipping plays an important role for those buyers to assess sellers; for example:

Very nice. The customer service representative is very patient. I finally feel relief after this test. One line [appeared on the reagent paper as a negative result] and there is not any [other] problem.

Shipping speed is fast. Operation is easy and [test result is] accurate. The package was delivered in discreet shipping.

This time of torture is finally over. I will definitely love my life after this. [AIDS] is such terrible. The reagent paper is good. The package [of HIVST kits] was handled in discreet shipping. This seller is considerate and the speed of delivery is fast.

As for the user experience of administering HIVST, there are several positive comments on the accuracy of the test and the speed of getting the result. The following are several examples:

Reagent paper has good quality. The test result is very accurate. Administering this test at home is so convenient.

Very good to check health status. I have been worried for several days and finally I feel relief to work. It’s so frightening. Reagent paper is nice and operation is convenient. I conducted [this test] really quick.

The quality is guaranteed. Safe and effective. The package from this seller is complete. The most important is to get the result fast and conveniently. [I] don’t have to worry about this every day!

Previously I used HIVST kits of other brands, but I still felt unsure [about test results]. Then I found this [brand name of HIVST kits] and knew many hospitals and disease control centers all used this [brand]. I also knew their HIVST kits got certified from World Health Organization PQ, which makes me trust the accuracy of test results. Thank [this brand] for producing such excellent kits.

Accurate, formal, safe, and convenient, but the lancet to collect blood samples is too short to use. It took several times to push blood out. Maybe it’s my own problem.

Interestingly, few commenters mentioned visiting a clinical center for conventional voluntary counseling and testing (VCT) to ensure their test results are accurate after obtaining negative results from HIVST. There are also few comments questioning the accuracy of the test result, which is not consistent with the previous findings that MSM in China were worried about the accuracy of HIVST [18,22]. One study found that MSM in China recognized HIVST as a supplement rather than a replacement for facility-based testing [12]. It remained a question as to whether the general HIVST kit users in China recognize HIVST as a replacement for facility-based testing methods without questioning the testing accuracy. Similar to the aforementioned quotation where a commenter took the testing accuracy of HIVST kit for granted because of its certificate from the World Health Organization Prequalification, most HIVST kit users did not express their concerns with operation errors and misinterpretation yielding negative test results, not to mention seeking VCT from clinic centers to ensure their test results.

There are also a few negative comments on sellers and user experiences. Some comments complained about the shipping speed, customer service, and the quality of tools inside HIVST kits. For example, one user complained about the shipping speed and finally gave up on waiting for the HIVST kits ordered on the web:

Shipping is a little bit slow. I ordered [HIVST kits] on Monday night…and received it on Thursday afternoon. I could not wait for that and went to a clinic (to test), which cost me 40 Chinese Yuan and 2 hours to get the result.

Another buyer posted a negative comment on a customer service representative who denied the problem caused by the quality of kits:

Thumb down (for this product). [Reagent paper] did not absorb the collected blood sample. [The customer service representative] said this was due to my operation error. I bought HIVST kits from many [other] sellers and I bought kits from this seller several times. It is the first time to have this problem…Please do not buy [HIVST kits] from this seller.

Several buyers commented on the lancet used to collect blood samples, such as biosafety of using such a lancet and how hurtful it would be. Zhang et al [22] reported similar observations in which MSM were worried that needles or lancets in HIVST kits were used by other people carrying HIV. A similar result is observed in this study:

One of the lancets looks like broken. Don’t know whether or not it was used by someone else. I was so scared.

#### Reasons for Buying HIVST Kits

Commenters also provided their reasons about why they decided to purchase and use HIVST kits. The reasons included risky sexual activities, nonsexual transmission, pure AIDS phobia, and preventive purposes. Most of the reasons are related to commenters’ own or their sexual (ex-) partners’ risky sexual activities, which made them purchase HIVST kits, for example:

Thanks! Heavens gave me a chance for a new life. I met a lady…and after that I got neck and spine pain with slight fever latterly. I saw AIDS prevention campaigns in my community and I found I got similar symptoms. I felt so scared. My brothers and sisters, please live a clean and moral life! I would not behave like that after this lesson. At last, thanks for this HIVST kit to bring me a new life.

It is necessary to test. Nowadays young people do not have only one partner. This disease has a long incubation period so it is necessary to test.

Don’t date young adults anymore in the future. It’s scary. Thank heavens for sparing my life. I will behave myself and I must use a condom. Safety comes first, my sisters.

I was once got drunk and had sex with someone. I was so scared but don’t have the courage [to get test]. Thank heavens for sparing my life. I will not drink alone in the future. I will live a clean and moral life.

There are also reasons focusing on using HIVST because of fear of nonsexual HIV transmission via blood and oral fluids; for example:

I was having a meal with a friend and was kissed suddenly [by this friend]. I felt so scared and decided to get a [HIV] test. I knew he went out for prostitution before. I went to a disease control center for counseling, and the doctor there was stubborn and did not give me any clear suggestions. Then I went to a hospital that specializes in infectious diseases for counseling. The nurse there told me HIV could not be transmitted via kissing, even tongue kissing, and suggested that I seek psychological therapies if I was so fearful.

While I was giving an injection to someone who might be a drug addict, [this person’s blood sample or oral fluids (not specified in the comment)] dropped on my fingernail. On the second day, it was found out that this [patient] got AIDS. Although there was no open wound on my skin, I was scared to death. I am a pregnant woman and I am so angry. I hope all AIDS patients should not hide their status while visiting a clinic. This is really dangerous for health practitioners. It’s harmful for both others and patients themselves.

Negative. Thank heavens for sparing me. I went to a small beauty salon to remove pimples a few years ago. I was so frightened since the needle used in this beauty salon might not get disinfected. Listen to me, if you want to do some cosmetic surgeries like ear piercing or eyebrow tattoo, you definitely should go to a formal beauty salon. Otherwise, you might have to worry about getting AIDS.

Clearly, these commenters know of nonsexual HIV transmission on some level, and they may have legitimate concerns to worry about getting HIV infection via medical tools or blood. However, their self-perceived likelihood of getting infected might be overestimated because of their misunderstanding of HIV transmission. They knew the ways of transmission but did not have a clear picture of the likelihood of getting HIV infection for each type of transmission and other HIV information such as how long HIV will be infectious outside the human body, which finally caused their panic and phobia and the buying of HIVST kits. The contagiousness of HIV was exaggerated, which is consistent with the previous study [39].

There are also a few cases in which HIVST kit users tested themselves because of AIDS phobia elicited by the popular science of HIV or they chose to test for preventive purposes, for instance:

I was told getting tooth extraction can even cause AIDS from popular science [articles or videos (not specified in the comment)]. I will get a premarital examination really soon. I was scared so I bought HIVST kits to test myself. I was so nervous before testing. My head was buzzing. I wondered what I should do if I really got [infected by HIV]? I put so much effort into dating a good boyfriend right now and I was afraid that [he] would leave me [because of AIDS]. Later, I realized that I just thought too much… Thank heavens for sparing my life. Although I did not fool around, I am neither homosexual nor a drug addict. However, it is still risky [to get HIV] from wounds if you do not use any bandage. As someone on the Internet said: AIDS-phobia makes me live a discreet life. Same as me.

Interestingly, this commenter did not engage in any risky behaviors and did not contact anyone who might carry HIV. She chose to take HIVST only because she was frightened by the popular science of HIV. She wanted to ensure her HIV status to prevent any possible embarrassment and loss before her marriage. This commenter even suggested that AIDS phobia helped her live a discreet life.

The aforementioned examples also demonstrated the stereotypes about people who live with HIV or AIDS, such as portraying people who live with HIV or AIDS as homosexual or drug addict. It raises a question about the effectiveness and the side effect of the popular science of HIV and people who live with HIV or AIDS, which may lead to further HIV-related panic, fear, and stigma.

In addition to these 4 main themes, there are some minor themes that did not appear frequently from these comments. For instance, there are a few comments concerning the misinformation and rumors about HIV or AIDS that they searched on the internet. One commenter said: “I will suggest to everyone that they should not check [a Chinese-based search engine] because [information searched on this site] frightened me a lot.” Another commenter was so scared by the misinformation on the internet about HIV and was not able to get a test immediately:

I found AIDS symptoms on the Internet matched with mine perfectly. I had been panicking for almost a whole year. I was too scared to get a test. I could only get comfort by telling myself that the chance [of getting HIV infection] is so slight via searching information on the Internet. However, it was said that the more you know, the more you fear. If you search [HIV] on [a Chinese-based search engine], you find yourself getting an incurable disease.

There is also a comment concerning the effect of using HIVST on mental health: “Just a suggestion, make sure you are psychologically prepared before purchasing [HIVST kits].” This is consistent with one of the drawbacks of taking HIVST that HIVST kit users receive little professional and appropriate psychological support and counseling in a nonclinical setting [4,13,22].

As a result, 4 main themes were identified from HIVST kit users’ comments. The first theme is concerned with the expression of positive and negative emotions. Many commenters under this theme thanked the heavens for sparing their life and showed their relief after receiving negative test results. Other commenters under this theme expressed hope for everyone else to receive negative test results and stay healthy. In addition, this theme is also characterized by commenters’ panic and fear of HIV infection before receiving negative test results and commenters felt extremely uncertain and unsafe about their future life interrupted by potential HIV infection. The second theme is concerned with living a clean and moral life in the future. Commenters under this theme not only expressed their determination to live a careful life but also offered sincere suggestions for others not to be sexually promiscuous around in the future. The third theme focuses on the evaluative comments on either sellers for their services, shipping speed, discreet shipping, conveniences, and other advantages over visiting clinics or HIVST kits for their quality. The fourth theme focuses on commenters’ reasons about why they chose to purchase HIVST kits. Most of the reasons reflected commenters’ fear of HIV infection by sexual transmission. Other reasons focused on nonsexual HIV transmission via blood or unclean medical tools. The implications for these findings are explained in the Discussion section after the results of the content analysis.

The first thematic analysis study explored some features of HIVST kit users’ comments posted on e-commerce platforms. However, owing to the inductive nature of the thematic analysis, this study only provides a basic picture of users’ concerns with a limited data sample. More systematic observations are needed to provide quantitative data about the frequency of each thematic feature based on a larger sample of comments. In addition, the sample yielding these 4 main themes was based on the mixed comments from 2 different e-commerce platforms, so the possible differences in these themes between platforms were not observed in this study. We will address these aforementioned questions in the second study of the quantitative content analysis based on 1857 comments on HIVST kits.

### Study 2: Quantitative Content Analysis

#### Overview

The second study provides quantitative data about frequencies of different thematic features of comments based on a larger sample of comments. In addition, we aimed to explore any potential differences regarding HIVST kit users’ comments between different e-commerce platforms. We conducted a quantitative content analysis of 1857 HIVST kits users’ comments posted on Tmall (n=975) and Pinduoduo (n=882). These 1857 comments were randomly selected from the entire data pool including 21,018 comments retrieved in December 2021. Regarding the random selection process for the content analysis, a Microsoft Excel add-in named Fangfanggezi [40] that enables random sampling within Excel was used. Quantitative content analysis was adopted for this study because it is widely accepted for studying buyers’ feedback comments [41,42] to understand patterns of a large-sized sample of messages.

#### Codebook Development

The codebook was developed based on the main themes identified in the first study. Different coding categories were elaborated upon based on thematic features under each theme including gratefulness, positive emotions (eg, relief and happiness) other than gratefulness, negative emotions, hope for negative test results and good health, calling for living a clean and moral life in the future, comments for sellers, comments for HIVST kits, and the reasons for buying HIVST kits. In addition to these coding categories developed from thematic features mentioned in the first study, there is one newly added coding category, users’ disclosures of their self-testing results, which emerged inductively from coders’ training processes. A single coding unit could be coded for multiple thematic features mentioned in the codebook. Details of the coding categories are presented in Table 1.

**Table 1:** Coding categories for quantitative content analysis.^a^

Category (thematic features)	Description of each level within the category^b^	Examples^c,d^
Gratefulness	1=commenters expressed gratefulness after receiving negative test results	“Thank heavens for sparing my life.” (Coded as 1) “Thanks. Heavens give me this opportunity. I will live a sexually abstinent life in the future. Being negative forever.” (Coded as 1)
Positive emotions other than gratefulness	1=commenters’ positive emotions (eg, relief and happiness) except from gratefulness	“I used it but I am not sure about the accuracy of the test result. Anyway, I feel much better now.” (Coded as 1) “Feel relief.” (Coded as 1)
Negative emotions	1=commenters’ negative emotions such as fear and guilt	“I heard about AIDS every and it scared me to death. In the future, I must live a sexually abstinent life.” (Coded as 1) “Finally, I don’t need to have my heart in my mouth any longer. The food today is so delicious.” (Coded as 1)
Hope for negative test results and good health	1=commenters expressed their hope for everyone receiving negative test results and staying healthy	“Wish everyone [getting] negative [test results]...” (Coded as 1) “Hope being negative forever. Hope everyone to stay healthy!” (Coded as 1)
Calling for living a clean and moral life in the future^e^	1=commenters called themselves to live a clean and moral life 2=commenters called others to live a clean and moral life 3=commenters called both themselves and others to live a clean and moral life 4=commenters encouraged others to get HIV tests but did not mention living a clean and moral life 5=keep on fooling around	“Thanks. [Hope] negative always. Keep living a sexually abstinent life. [Live] healthy and happily.” (Coded as 1) “Dear strangers. Promise me. Love yourself.” (Coded as 2) “Live a sexually abstinent life, bros.” (Coded as 2) “Thank heavens for giving me an opportunity of the rebirth. I must live a sexually abstinent life in the future. And hope other bros to live a sexually abstinent life.” (Coded as 3) “I can continue to fool around...” (Coded as 5)
Comments for sellers	1=positive evaluation for sellers (regarding convenience, shipping speed, discreet shipping, price, customer service, etc) 2=Negative evaluation for sellers	“It is the discreet shipping. Shipping speed is fast...” (Coded as 1) “First time I bought two kits...now I finally got off from this fear. Thank the seller for their patient explanations.” (Coded as 1) “Customer service representative No. XXX is very irresponsible!!!...Good attitude before the purchase but attitude changed after the purchase!!!...When the customer had a problem which needed her explanation, she was not patient! Being distracted!!!...” (Coded as 2)
Comments for kits	1=positive evaluation for kits (regarding testing accuracy, biosafety, and easiness of operation and interpretation, etc) 2=Negative evaluation for kits	“Safe and convenient. Good attitudes for customer service.” (Coded as 1) “The reagent paper is very good and convenient. Thank the boss.” (Coded as 1) “The lancet is broken and could not be used. Please don’t buy it everyone.” (Coded as 2) “Useless. I doubt if this thing can test (the disease) at all.” (Coded as 2) “Garbage. The reagent paper is useless...” (Coded as 2)
Reasons	1=commenters’ uptake of HIVST^f^ because of pure phobia 2=commenters’ uptake of HIVST because of their or their (ex)partners’ risky sexual activities 3=commenters’ uptake of HIVST because of nonsexual transmission 4=commenters’ uptake of HIVST because of feeling bored or curious 5=commenters’ uptake of HIVST for preventive purposes	“I must be crazy. I did nothing. Once the teacher mentioned this in class, I felt panic. I bought the kit and it’s nothing wrong. I just want to feel relief. In the future, I shall no longer look at these websites about hiv...” (Coded as 1) “I had two tests and both were negative. In the future, I must stay away from the scumbag guy. Thank the seller.” (Coded as 2) “I have tattoo. It was said the tattoo needles are not clean. I was scared so I bought the kit to test as soon as I can...It is best that you don’t get tattoo or teeth extraction if there isn’t any problem. It saves your time for worrying about yourself.” (Coded as 3) “Bought this for my curiosity.” (Coded as 4) “It is necessary for adults to take the test regularly. Very good, easy to use, and convenient.” (Coded as 5)
Disclosures of self-testing results	1=there is enough evidence to infer that the user received a negative test result (eg, Thank heavens for sparing my life!) 2=there is enough evidence to infer that the user received a positive test result 3=commenters felt unsure about results	“Being worried for a few days. I received [the kit] and get self-testing. There is not any problem at all. I can feel relief now.” (Coded as 1) “Positive results twice...” (Coded as 2) “I am not sure whether the test result is accurate. The operation is convenient.” (Coded as 3)

^a^All coding categories are not mutually exclusive. Krippendorff α values for all categories ranged from .79 to 1.00.

^b^0=absence of a particular category.

^c^We corrected typos and grammar errors.

^d^Examples were selected from 1857 comments for the content analysis.

^e^There are several newly added levels within a category such as levels 3, 4, and 5 in the category of calling for action. They were included during the training process as suggested by 3 coders.

^f^HIVST: HIV self-testing.

#### Coder Training and Procedures of Analysis

Three coders were trained using the codebook to code 50 comments to test the codebook’s clarity and ensure their understanding of it. Then, each of the 3 coders were provided the same set of 50 new comments to code independently. After they completed coding, intercoder reliabilities between them were calculated, and the coders were brought together to discuss and reconcile their discrepancies in terms of coding. Their feedback was also used to modify the coding categories in the codebook. This procedure of independent coding was repeated thrice. One new coding category of disclosing HIVST results mentioned earlier was included in the original codebook during these 3 trials. The training finally yielded accepted intercoder reliability (Krippendorff α values ranged from .79 to 1.00 for all 9 coding categories). Then, each of the coders was provided a different set of about 600 new comments to code independently, yielding 1857 comments in total for the quantitative content analysis.

#### Frequencies of Thematic Features in Total

The frequencies of different thematic features in total and in each platform are displayed in Table 2. The top-5 predominant thematic features in total (N=1857) included comments for HIVST kits (906/1857, 48.79%), disclosures of self-testing results (869/1857, 46.79%), comments for sellers (593/1857, 31.93%), gratefulness (493/1857, 26.55%), and calling for living a clean and moral life (341/1857, 18.36%). It is not surprising to find that the predominant thematic features observed are related to evaluations for HIVST kits and sellers since the data were based on feedback comments on e-commerce platforms. Surprisingly, there are many comments disclosing their self-testing results. HIVST kit users may consider e-commerce platforms as an anonymous stage to express their concerns and discuss their test results without worrying about HIV stigma. However, only a few commenters disclosed their positive test results (6/1857, 0.32%). Most commenters reported negative test results (833/1857, 44.86%). It is questionable whether those receiving positive test results may feel more reluctant to disclose their status in their feedback comments because of HIV stigma. On the other hand, even though most commenters received negative test results, their acts of disclosing the test results and (more importantly) the fact that they conducted HIVST implied their HIV awareness, self-efficacy, and courage to counter HIV stigma instead of regarding the taking of HIV tests as a controversial and stigmatized topic. In addition, regarding the comments for sellers and kits, there were more positive evaluations than negative evaluations, which suggested that, at least for HIVST kit users, most kits sold on the web met their needs.

**Table 2:** Frequencies for thematic features in total and each platform (N=1857; Tmall: n=975; Pinduoduo: n=882).

Thematic features			Total, n (%)		Tmall, n (%)		Pinduoduo, n (%)
Gratefulness^a^			493 (26.5)		206 (21.1)		287 (32.5)
Positive emotions other than gratefulness			184 (9.9)		97 (9.9)		87 (9.9)
Negative emotions			251 (13.5)		134 (13.7)		117 (13.3)
Hope for negative test results and good health			71 (3.8)		38 (3.9)		33 (3.7)
**Calling for living a clean and moral life in the future^b^**			341 (18.3)		139 (14.2)		202 (22.9)
	Calling themselves to live a clean and moral life	310 (16.7)		124 (12.7)		186 (21.1)	
	Calling others to live a clean and moral life	19 (1)		11 (1.1)		8 (0.9)	
	Calling themselves and others to live a clean and moral life	10 (0.5)		2 (0.2)		8 (0.9)	
	Keep on fooling around in the future	2 (0.1)		2 (0.2)		0 (0)	
**Comments for sellers^a^**			593 (31.9)		291 (29.9)		302 (34.3)
	Positive evaluation	562 (30.2)		266 (27.3)		296 (33.6)	
	Negative evaluation	31 (1.7)		25 (2.6)		6 (0.7)	
**Comments for HIVST^c^ kits^a^**			906 (48.8)		470 (48.2)		436 (49.4)
	Positive evaluation	856 (46.1)		430 (44.1)		426 (48.3)	
	Negative evaluation	50 (2.7)		40 (4.1)		10 (1.1)	
**Reasons**			290 (15.7)		156 (16.1)		134 (15.1)
	Pure phobia	133 (7.2)		78 (8)		55 (6.2)	
	Risky sexual activities	85 (4.6)		30 (3.1)		55 (6.2)	
	Nonsexual transmission	29 (1.6)		21 (2.2)		8 (0.9)	
	Feeling bored or curious	9 (0.5)		6 (0.6)		3 (0.3)	
	Preventive purpose	34 (1.8)		21 (2.2)		13 (1.5)	
**Disclosures of self-testing results**			869 (46.8)		417 (42.8)		452 (51.2)
	Negative	833 (44.9)		393 (40.3)		440 (49.9)	
	Positive	6 (0.3)		4 (0.4)		2 (0.2)	
	Unsure about results	30 (1.6)		20 (2.1)		10 (1.1)	

^a^A significant association between this thematic feature and platforms (*P*<.001).

^b^There is level 4 under the category of “calling for living a clean and moral life” in which commenters encouraged others to get HIV tests but did not mention living a sexually abstinent life (Table 1). This level was added during the training process based on the training data, but there were not any comments in the main study which can be coded for this level. Hence, it was removed from Table 2.

^c^HIVST: HIV self-testing.

#### Differences Between Tmall and Pinduoduo

The relationship between each one of the thematic features and e-commerce platforms were examined by using Chi-square tests to explore possible platform differences. Some significant results were observed. The association between the thematic feature of gratefulness and platforms was significant (N=1857, χ^2^_1_=30.9, *P*<.001). The association between comments for sellers and platforms was significant (N=1857, χ^2^_2_=17.2, *P*<.001). The association between comments for HIVST kits and platforms was also significant (N=1857, χ^2^_2_=17.1, *P*<.001). Table 2 presents details on the platform differences.

#### Other Findings

Chi-square tests were also conducted to examine the relationships between different types of thematic features, and some significant relationships were observed. Gratefulness was associated with multiple other themes: positive emotions other than gratefulness (N=1857, χ^2^_1_=90.7, *P*<.001); negative emotions (N=1857, χ^2^_1_=67.3, *P*<.001); comments for seller (N=1857, χ^2^_2_=53.8, *P*<.001); comments for HIVST kits (N=1857, χ^2^_1_=202.6, *P*<.001). In addition, the theme of negative emotions was associated with positive emotions other than gratefulness (N=1857, χ^2^_1_=25.3, *P*<.001); the theme of negative emotions was also associated with comments for HIVST kits (N=1857, χ^2^_1_=76.7, *P*<.001).

The content analysis of the comments on HIVST kits from the 2 popular e-commerce platforms in China yielded significant differences in thematic features across the platforms. Some significant relationships between the themes themselves were also observed. The frequencies for thematic features also provided a clear picture of predominant features observed in these comments. The implications of these findings are explained in the Discussion section.

## Discussion

### Principal Findings

On the basis of the infodemiological approach, both studies yielded important findings on Chinese HIVST kit users’ concerns and health awareness across 2 e-commerce platforms. To the best of our knowledge, this is the first study examining the feedback comments of web-based HIVST kit users. The qualitative thematic analysis identified 4 themes featuring users’ emotional responses before and after self-testing, behavioral intentions (eg, living a clean and moral life), evaluations for sellers and kits, and reasons for purchase. The quantitative content analysis provided the frequencies of different thematic features, explored platform differences of these features, and examined the relationships between thematic features. The implications of both studies are discussed in subsequent sections.

### Thematic Analysis

On the basis of the results of the first thematic analysis, many commenters expressed their gratefulness and relief for receiving negative results, but few positive-result comments were observed. This theme features HIVST kits users thanking the heavens for sparing their life and giving them rebirth. For these commenters, getting an HIV infection depends on the heavens instead of themselves. This result also implies HIV fatalism, which is the belief that HIV acquisition and mortality is beyond one’s own control [43]. Such a fatalistic attitude might become an obstacle for health promotion and HIV prevention campaigns since HIV fatalism is also associated with risky sexual activities [44,45].

Interestingly, few comments addressed the importance of using condoms before engaging in risky sexual activities, but many comments emphasized the importance of living a clean and moral life in the future (theme 2). This calling for living a better life suggests HIVST kit users, although being influenced by HIV fatalism on some level, still believe that they can control their health in the future by living a moral life. This contradicting phenomenon might result from Chinese moral beliefs based on Confucianism and Buddhism [46] that misbehaviors bring about punishments from the heavens and correcting misbehaviors bring about the removal of punishments from the heavens. Confucianists believe that one’s destiny is determined by their moral efforts and that negative outcomes are the consequences of moral failures; Buddhists, on the other hand, believe that good acts will earn positive outcomes and bad acts will lead to negative repercussions [46]. Chinese virtues emphasizing sexual purity tend to equalize promiscuous lifestyles, infidelity, and prostitution as a sin and moral failure (see the study by Zhai [47] for details on Chinese virtues about sexual purity). Getting an HIV infection via risky sexual activities is the sign of punishment from the heavens for committing a sex-related sin. Therefore, those who expressed thanks to the heavens may consider themselves sinful or as failing morally for their debauched and promiscuous lifestyles, so their HIV status depends on the heavens. If they promise to the heavens to live a clean and moral life in the future, then the punishment of an HIV infection is preventable or at least controllable due to their moral efforts. Future studies should explore how to emphasize self-efficacy to prevent HIV instead of equalizing HIV or AIDS as a sin or punishment due to moral failures.

In addition, few commenters mentioned seeking VCT to ensure their test results are correct, and there were few doubts about the accuracy of testing results. It is plausible that commenters felt no need to confirm since they had received negative results. However, there is also a risk of misoperation or misinterpretation of results, not to mention the poor quality of uncertified kits or kits damaged during shipping that may also result in accurate results. Although previous studies suggested that the consistency between HIVST results and antibody detection results was 90.5% [7], and most participants could perform the self-test correctly and obtain accurate test results at home [48], there is always a risk of a false negative. Many HIVST users did not receive appropriate education on the limitations of HIVST kits. The relief HIVST kit users expressed in the first theme might just be seeming relief without confirmation from formal VCT centers. Some comments also showed a misunderstanding of HIV transmission. The results suggested that more HIV and AIDS education campaigns are needed.

### Content Analysis

The results of content analysis showed that a large number of commenters expressed their gratefulness suggesting these commenters considered that whether they get HIV is not fully controllable but dependent on some spiritual factors outside of their control. Therefore, there is a need to understand the underlying reason why these people like to take their chances rather than refrain from risky activities. The examination of the reasons for buying HIVST kits revealed that the motivations behind HIVST self-testing are mostly pure AIDS phobia or related to risky sexual activities. Many commenters indicated that they suspected they might get infected after watching an AIDS-related video on a short video app. On the one hand, such media-induced AIDS phobia is an effective way to increase HIVST uptake in the general population. On the other hand, media-induced AIDS phobia could cause anxiety and worry, leading to long-term emotional distress. In addition, the percentage of pure phobia reasons is higher than risky sexual activities reasons. One possible explanation is that many Chinese buyers feel uncomfortable sharing their sexual experiences on the web because people do not usually talk about sex publicly in traditional Chinese culture [47]. It is reasonable to expect that there were many more people who took HIVST because of their previous engagement in risky sexual activities as there is a high percentage of comments calling themselves to live an abstinent life. Apart from pure phobia and risky sexual activities, there are other reasons such as preventive purposes and fear of nonsexual transmission.

Nearly 50% of the comments were related to the product itself and 32% were related to the seller. It suggested that most HIVST kit users were concerned with the quality of the product and services that the seller provided. Interestingly, it was observed that many users commented that “the test result is very accurate,” although they might not have the result from the hospital to compare with. Such a comment reflected the users’ sincere hope for a negative result. There were more positive comments (n=562) than negative comments (n=31) for sellers. Similarly, for HIVST kits, more positive comments (n=856) were observed in the data than negative comments (n=50). It is plausible that Chinese users in our data sample felt reluctant to report a negative user experience or that some positive feedback reviews were fabricated. Some firms pay people to write a fake review using fake identities on the web [49], so the numbers and frequencies of positive comments on sellers and kits might be overly estimated.

There were some significant differences between Tmall and Pinduoduo data. For example, the percentage of expressing gratefulness in comments is higher for Pinduoduo than Tmall. One explanation is that there are user differences. The user portrait of Pinduoduo is notably different from Tmall as a large portion of Pinduoduo users are older adults looking for affordable products in small cities [50], and most Tmall users are between the ages of 30 and 39 years who come from first-tier cities [51]. In addition, some thematic features were significantly related to other thematic features. For example, gratefulness was related to comments on sellers, comments on HIVST kits, positive emotions other than gratefulness, and negative emotions. Similarly, negative emotions were related to positive emotions other than gratefulness and comments on HIVST kits. This may suggest that expressing feelings of gratefulness and negative emotions might be an indicator for expressing other types of emotions and feedbacks. The findings might also imply that the HIVST kit users’ emotional status was related to their evaluation on products. Gratefulness felt by these users, as a positive emotion, was related to users’ evaluation on both sellers and products. Negative emotions, on the other hand, were related to the evaluation on products rather than sellers. Future studies may consider conducting sentiment analysis to understand the contextual meaning of feedback comments of HIVST kits. In addition, future studies might adopt the survey method to measure HIVST kit users’ gratefulness and other emotions, their evaluation of products and sellers, their intention to live a clean and moral life in the future, and their acts of disclosing self-testing results in feedback comments on platforms.

### Implications and Future Directions

Theoretically, this study explored Chinese cultural influences on attitudes toward HIV prevention and acquisition. Although comments such as “thank heavens” implied HIV fatalism that HIV acquisition is out of one’s own control. Many commenters believed they should live a discreet and virtuous life in the future since the heavens had decided to spare their life. This implied a traditional Chinese health belief in which health is influenced by one’s morality [46]. This belief may result in health-related stigma leading to fear of moral contamination and of losing face for people who carry such health stigma [23,52]. People who live with HIV or AIDS are stigmatized as immoral and indecent [39]. Some HIVST kit users in this study carried the same moral judgment toward people who live with HIV or AIDS and HIV self-stigma when suspecting themselves of getting infected by HIV. However, by cultivating morality via calling for living a clean and moral life in the future, HIVST kit users believe that they still have control over their health to prevent HIV. Public health scholars, practitioners, and policy makers should explore how this traditional Chinese health belief relating morality to health still influences contemporary Chinese users’ responses to HIV infection and prevention. More specifically, does this health belief elicit HIV stigma or fatalism in China? Or does this health belief help to strengthen people’s agency to prevent HIV? Future studies should consider analyzing HIVST kit users’ feedback comments regarding their self-stigmatization, concerns with face loss, feelings of shame, and fear of moral contamination threatening to themselves and their family members.

Methodologically, relying on authentic user-generated information from a more general population and the multimethod approach, this paper heralded a new direction for HIVST research in China. Previous HIVST studies in China mostly focused on MSM populations and heavily relied on obtrusive methods such as surveys [18,53] and interviews [12,22]. By analyzing user-generated information from 2 e-commerce platforms, this paper avoided possible bias caused by obtrusive methods such as social desirability bias. In addition, by adopting the multimethod approach and the infodemiological perspective, this study provided a detailed picture of HIVST kit users’ authentic concerns, which were rarely addressed in previous HIVST studies in China. It is recommended that future studies should consider adopting machine learning techniques or the Linguistic Inquiry and Word Count-22 to analyze more feedback comments of HIVST kits on e-commerce websites. Techniques detecting fake reviews should be incorporated in a future study to obtain a more accurate estimation of positive evaluations on sellers and HIVST kits. There were few commenters disclosing a positive test result in the data, future studies should consider how to use unobtrusive methods to obtain the information about their responses. For example, HIVST kit users who got a positive test result might seek help from customer service representatives to ensure their results are accurate. Future studies may also consider interviewing the customer service representatives of HIVST kits about their communication with HIVST kit users to know the latter’s inquiries and concerns. In addition, little is known about how these HIVST kit users read the manual included in the kit package and how they conducted testing based on feedback comments. These might be addressed by either interviewing customer service representatives or HIVST kit users themselves about the difficulties the users encountered during self-testing.

Practically, this study raised a few concerns with the uptake of HIVST in China. Both studies indicated that only a few Chinese users questioned the quality of HIVST kits based on the feedback comments of top-selling HIVST kits sold on popular e-commerce platforms. In addition, very few commenters mentioned visiting hospitals to confirm their test results. There are still possible errors due to misoperation and misinterpretation while taking HIVST. It is crucial to incorporate counseling and confirmation services into HIVST business to avoid potential false reassurances caused by HIVST. In addition, the results of this study suggested that HIVST kit users were overwhelmed by misinformation on the web about HIV transmission, infection, and symptoms, which led to some users’ AIDS phobia. Future public health campaign designers should consider including brochures or flyers with credible HIV-related knowledge inside HIVST kit packages.

### Limitations

This study has its limitations. First, the results might be subject to sampling bias as the buyers whose HIVST results were positive felt reluctant to share their results on the web. Only 0.32% (6/1857) of the commenters shared their positive results in the content analysis. In the same vein, the results of the reasons category may not represent the whole picture as only a small percent of commenters shared the reason why they took HIVST. Second, the comments analyzed in this study was based on the most recent comments on December 2021. It is hard to know whether an extended data collection period would yield different results. The comments analyzed in this study were based on the top-selling products from 2 e-commerce websites and may not be representative of the whole HIVST products sold in China. Such limitations may raise some concerns with the external validity of the results. Third, although platform differences were observed, there is a lack of users’ demographic information for further analysis due to methodological limitations of analyzing users’ feedback comments. Fourth, it is difficult to distinguish the comments based on users’ authentic experiences from intentionally produced fake reviews posted by sellers or competitive sellers [49]. Many duplicated comments generated by the same commenters posted during the same period were observed in retrieved Pinduoduo data. These comments were doubted for being fake reviews and were removed from the entire data corpus. Although we took some procedures to remove potentially fake reviews in the data, there is no guarantee that all kept comments were authentic. Future studies should consider adopting fake-review detection techniques such as developing algorithms based on big data from the social platforms or adopting sentiment analysis of written comments [54] to remove fabricated feedback comments. After removal of the fake comments, future studies may consider adopting purposive sampling methods to only examine the relevant comments related to HIVST kit users’ certain experiences such as the emotional responses after getting negative test results or their complaints on kits. Fifth, several HIVST kits were sold as part of kit packages including different types of self-testing kits for detecting various diseases. Some packages include the HIVST kit and the kit to test hepatitis B or syphilis (which also requires a blood sample to conduct self-testing similar to HIVST). Because the transmission of hepatitis B and syphilis is similar to HIV transmission, it is difficult to ensure that all the comments were targeted at HIVST kits alone. On the other hand, among 1857 comments analyzed in this study, only 3 mentioned hepatitis B and only 8 mentioned syphilis. Hence, it is assumed that most comments were still concerned with HIV and AIDS while future studies should consider how to distinguish comments specifically targeting HIVST from other comments. Sixth, all comments were retrieved from either the Tmall mobile app or the Pinduoduo mobile app. While Pinduoduo is a mobile-only platform, Tmall has both mobile and website platforms, and comments from both Tmall platforms were mixed together. Future studies should explore how the mobile and website difference influences the content of feedback comments. Finally, when users enter feedback comments on Pinduoduo, some pop-up words are suggested by this platform that are displayed next to the comment section, such as “good quality and low price.” If users click these pop-up words, the words will be automatically entered as a part of feedback comments. It is hard to evaluate the authenticity of certain comments that have the same content because of this platform feature, and future studies may consider how to detect these comments.

## Abbreviations

HIVST: HIV self-testing
MSM: men who have sex with men
VCT: voluntary counseling and testing

## References

[1] Li G, Jiang Y, Zhang L (2019). HIV upsurge in China's students. Science.

[2] Ritchwood TD, Selin A, Pettifor A, Lippman SA, Gilmore H, Kimaru L, Hove J, Wagner R, Twine R, Kahn K (2019). HIV self-testing: South African young adults' recommendations for ease of use, test kit contents, accessibility, and supportive resources. BMC Public Health.

[3] Koris AL, Stewart KA, Ritchwood TD, Mususa D, Ncube G, Ferrand RA, McHugh G (2021). Youth-friendly HIV self-testing: acceptability of campus-based oral HIV self-testing among young adult students in Zimbabwe. PLoS One.

[4] Johnson C, Baggaley R, Forsythe S, van Rooyen H, Ford N, Napierala Mavedzenge S, Corbett E, Natarajan P, Taegtmeyer M (2014). Realizing the potential for HIV self-testing. AIDS Behav.

[5] Wong HT, Tam HY, Chan DP, Lee SS (2015). Usage and acceptability of HIV self-testing in men who have sex with men in Hong Kong. AIDS Behav.

[6] Wong V, Johnson C, Cowan E, Rosenthal M, Peeling R, Miralles M, Sands A, Brown C (2014). HIV self-testing in resource-limited settings: regulatory and policy considerations. AIDS Behav.

[7] Liu Y, Wu G, Lu R, Ou R, Hu L, Yin Y, Zhang Y, Yan H, Zhao Y, Luo Y, Ye M (2020). Facilitators and barriers associated with uptake of HIV self-testing among men who have sex with men in Chongqing, China: a cross-sectional survey. Int J Environ Res Public Health.

[8] Brown 3rd W, Carballo-Diéguez A, John RM, Schnall R (2016). Information, motivation, and behavioral skills of high-risk young adults to use the HIV self-test. AIDS Behav.

[9] Mugo PM, Micheni M, Shangala J, Hussein MH, Graham SM, Rinke de Wit TF, Sanders EJ (2017). Uptake and acceptability of oral HIV self-testing among community pharmacy clients in Kenya: a feasibility study. PLoS One.

[10] Pant Pai N, Bhargava M, Joseph L, Sharma J, Pillay S, Balram B, Tellier PP (2014). Will an unsupervised self-testing strategy be feasible to operationalize in Canada? Results from a pilot study in students of a large Canadian University. AIDS Res Treat.

[11] Carballo-Diéguez A, Frasca T, Balan I, Ibitoye M, Dolezal C (2012). Use of a rapid HIV home test prevents HIV exposure in a high risk sample of men who have sex with men. AIDS Behav.

[12] Liu F, Qin Y, Meng S, Zhang W, Tang W, Han L, Liu C, Zhang Y, Huang S, Zheng H, Yang B, Tucker JD (2019). HIV self-testing among men who have sex with men in China: a qualitative implementation research study. J Virus Erad.

[13] Martinez O, Carballo-Diéguez A, Ibitoye M, Frasca T, Brown W, Balan I (2014). Anticipated and actual reactions to receiving HIV positive results through self-testing among gay and bisexual men. AIDS Behav.

[14] (2017). Thirteenth Five-Year Plan (2017-22) for HIV prevention and control. The State Council of the People’s Republic of China.

[15] (2022). The 24th World AIDS conference China satellite conference was successfully held. Fuzhou Centers for Disease Control and Prevention.

[16] (2019). The manual of AIDS self-testing guidance. Chinese Centers for Disease Control and Prevention.

[17] Han L, Bien CH, Wei C, Muessig KE, Yang M, Liu F, Yang L, Meng G, Emch ME, Tucker JD (2014). HIV self-testing among online MSM in China: implications for expanding HIV testing among key populations. J Acquir Immune Defic Syndr.

[18] Zhong F, Tang W, Cheng W, Lin P, Wu Q, Cai Y, Tang S, Fan L, Zhao Y, Chen X, Mao J, Meng G, Tucker JD, Xu H (2017). Acceptability and feasibility of a social entrepreneurship testing model to promote HIV self-testing and linkage to care among men who have sex with men. HIV Med.

[19] Yan H, Yang H, Raymond HF, Li J, Shi LE, Huan X, Wei C (2015). Experiences and correlates of HIV self-testing among men who have sex with men in Jiangsu province, China. AIDS Behav.

[20] Huang Z, Dai W, Zhou Y, Li X, Lin K, Jiang H (2020). Preference and related factors of HIV testing modes among men who have sex with men. Chinese J AIDS STD.

[21] Lu Y, He X, Ma J, Yao J, Xin W, Liu P, Jiang Y (2019). Online purchase of HIV rapid detection reagents for self-testing population characteristics. Chinese J AIDS STD.

[22] Zhang C, Li X, Heilemann MV, Chen X, Wang H, Koniak-Griffin D (2021). Facilitators and barriers of HIV self-testing among Chinese men who have sex with men: a qualitative study. J Assoc Nurses AIDS Care.

[23] Yang LH, Kleinman A (2008). 'Face' and the embodiment of stigma in China: the cases of schizophrenia and AIDS. Soc Sci Med.

[24] Blackstone A (2014). Principles of Sociological Inquiry: Qualitative and Quantitative Methods. Version 1.0.

[25] Eysenbach G (2002). Infodemiology: the epidemiology of (mis)information. Am J Med.

[26] Eysenbach G (2009). Infodemiology and infoveillance: framework for an emerging set of public health informatics methods to analyze search, communication and publication behavior on the Internet. J Med Internet Res.

[27] Eysenbach G (2011). Infodemiology and infoveillance tracking online health information and cyberbehavior for public health. Am J Prev Med.

[28] Manchaiah V, Amlani AM, Bricker CM, Whitfield CT, Ratinaud P (2019). Benefits and shortcomings of direct-to-consumer hearing devices: analysis of large secondary data generated from Amazon customer reviews. J Speech Lang Hear Res.

[29] Zeraatkar K, Ahmadi M (2018). Trends of infodemiology studies: a scoping review. Health Info Libr J.

[30] Lee A, Mmonu NA, Thomas H, Rios N, Enriquez A, Breyer BN (2021). Qualitative analysis of Amazon customer reviews of penile clamps for male urinary incontinence. Neurourol Urodyn.

[31] Braun V, Clarke V (2006). Using thematic analysis in psychology. Qual Res Psychol.

[32] Riffe D, Lacy S, Fico F (2005). Analyzing Media Messages: Using Quantitative Content Analysis in Research. 2nd edition.

[33] Walk-Morris T (2019). What is it about Tmall?. Retail Dive.

[34] (2017). 22 amazing Tmall statistics. DMR.

[35] CIW Team (2021). Pinduoduo’s active buyers totaled 867 million in Q3 2021. China Internet Watch.

[36] Shank GD (2005). Qualitative Research: A Personal Skills Approach. 2nd edition.

[37] Frasca T, Balan I, Ibitoye M, Valladares J, Dolezal C, Carballo-Diéguez A (2014). Attitude and behavior changes among gay and bisexual men after use of rapid home HIV tests to screen sexual partners. AIDS Behav.

[38] Zhang YX, Golin CE, Bu J, Emrick CB, Nan Z, Li MQ (2014). Coping strategies for HIV-related stigma in Liuzhou, China. AIDS Behav.

[39] Zhou YR, Liamputtong P (2013). Morality, discrimination, and silence: understanding HIV stigma in the sociocultural context of China. Stigma, Discrimination and Living with HIV/AIDS: A Cross-Cultural Perspective.

[40] FFCell.

[41] Qu Z, Zhang H, Li H (2008). Determinants of online merchant rating: content analysis of consumer comments about Yahoo merchants. Decis Support Syst.

[42] Pavlou PA, Dimoka A (2006). The nature and role of feedback text comments in online marketplaces: implications for trust building, price premiums, and seller differentiation. Inf Syst Res.

[43] Sileo KM, Bogart LM, Wagner GJ, Musoke W, Naigino R, Mukasa B, Wanyenze RK (2019). HIV fatalism and engagement in transactional sex among Ugandan fisherfolk living with HIV. SAHARA J.

[44] Hess RF, McKinney D (2007). Fatalism and HIV/AIDS beliefs in rural Mali, West Africa. J Nurs Scholarsh.

[45] Akande A (1997). Black South African adolescents' attitudes towards AIDS precautions. School Psychol Int.

[46] Lam C, Tsang HW, Corrigan P, Lee YT, Angell B, Shi K, Jin SH, Larson JE (2010). Chinese lay theory and mental illness stigma: implications for research and practices. J Rehabil.

[47] Zhai X (2011). Perspectives on Chinese “Face”: Psychological Motives and Social Representations.

[48] Ibitoye M, Frasca T, Giguere R, Carballo-Diéguez A (2014). Home testing past, present and future: lessons learned and implications for HIV home tests. AIDS Behav.

[49] Malbon J (2013). Taking fake online consumer reviews seriously. J Consum Policy.

[50] Graziani T (2019). Pinduoduo expands to Tier 1 cities. WalktheChat.

[51] (2018). Product Analysis Report-Tmall. Jianshu.

[52] Yang LH, Kleinman A, Link BG, Phelan JC, Lee S, Good B (2007). Culture and stigma: adding moral experience to stigma theory. Soc Sci Med.

[53] Marley G, Kang D, Wilson EC, Huang T, Qian Y, Li X, Tao X, Wang G, Xun H, Ma W (2014). Introducing rapid oral-fluid HIV testing among high risk populations in Shandong, China: feasibility and challenges. BMC Public Health.

[54] Reyes-Menendez A, Saura JR, Filipe F (2019). The importance of behavioral data to identify online fake reviews for tourism businesses: a systematic review. PeerJ Comput Sci.

